# Comparing the quality of pro- and anti-vaccination online information: a content analysis of vaccination-related webpages

**DOI:** 10.1186/s12889-016-2722-9

**Published:** 2016-01-15

**Authors:** Gabriele Sak, Nicola Diviani, Ahmed Allam, Peter J. Schulz

**Affiliations:** 1 Institute of Communication and Health (ICH), Università della Svizzera italiana (USI), Via G. Buffi 13, 6900 Lugano, Switzerland; 2 Amsterdam School of Communication Research / ASCoR, University of Amsterdam, Amsterdam, The Netherlands; 3 Department of Pathology, Yale University School of Medicine, New Haven, USA

**Keywords:** Online information quality, Comparative content analysis, Pro- and anti-vaccination webpages

## Abstract

**Background:**

The exponential increase in health-related online platforms has made the Internet one of the main sources of health information globally. The quality of health contents disseminated on the Internet has been a central focus for many researchers. To date, however, few comparative content analyses of pro- and anti-vaccination websites have been conducted, and none of them compared the quality of information. The main objective of this study was therefore to bring new evidence on this aspect by comparing the quality of pro- and anti-vaccination online sources.

**Methods:**

Based on past literature and health information quality evaluation initiatives, a 40-categories assessment tool (Online Vaccination Information Quality Codebook) was developed and used to code a sample of 1093 webpages retrieved via Google and two filtered versions of the same search engine. The categories investigated were grouped into four main quality dimensions: web-related design quality criteria (10 categories), health-specific design quality criteria (3 categories), health related content attributes (12 categories) and vaccination-specific content attributes (15 categories). Data analysis comprised frequency counts, cross tabulations, Pearson’s chi-square, and other inferential indicators.

**Results:**

The final sample included 514 webpages in favor of vaccination, 471 against, and 108 neutral. Generally, webpages holding a favorable view toward vaccination presented more quality indicators compared to both neutral and anti-vaccination pages. However, some notable exceptions to this rule were observed. In particular, no differences were found between pro- and anti-vaccination webpages as regards vaccination-specific content attributes.

**Conclusions:**

Our analyses showed that the overall quality of pro-vaccination webpages is superior to anti-vaccination online sources. The developed coding scheme was proven to be a helpful and reliable tool to judge the quality of vaccination-related webpages. Based on the results, we advance recommendations for online health information providers as well as directions for future research in this field.

**Electronic supplementary material:**

The online version of this article (doi:10.1186/s12889-016-2722-9) contains supplementary material, which is available to authorized users.

## Background

According to the International Telecommunication Union [1], currently almost 40 % of the global population (approximately 3 billion people) and 78 % of the developed world’s population is online. Thanks to the Internet’s persuasive force produced by the intersection of mass media and interpersonal communication elements, online health information seeking is becoming a recurrent activity of people’s everyday life [2–4]. The majority of US internet users who have gone online to retrieve health information searched for health contents related to a particular disease or medical problem, and as a second most frequent “surfing activity” they looked for web sources describing a specific medical treatment or procedure [4]. The same portion of internet information seekers reported that their online health session started via a general search engine such as Google [4]. As Internet health consumers are now able to get access to multiple sources of health information without much effort, their level of knowledge and their social roles in the health domain might be affected. Depending on the quality of the information retrieved, the latter can impact people’s attitudes toward a specific health topic and condition either beneficially or deleteriously.

The quality of health contents disseminated on the Internet has been a central focus for many researchers in the last decades. A systematic review showed that 70 % of 79 studies included found the general value of the information retrieved to be low, and another 20 % found it to be mediocre [5]. In response to criticism describing online health information as misleading, biased, highly technical, dated and fraudulent, different international and national bodies issued various codes of conduct in order to regulate and monitor the quality of health contents [6, 7], providing “a wide range of tools to assist site developers to produce quality sites and for consumers to assess the quality of sites” ([8] p. 598). Even though these protective initiatives often make use of rather similar quality criteria and set up similar ethical standards (e.g., disclosure of sources of information), their scope and application slightly differ [9]. These quality instruments can be grouped into five overarching types: “codes of conduct, quality labels, user guides, filters, and third party certification” [8]. If, on the one hand, this emphasizes the growing need to assess the value of online health information, on the other hand it highlights the lack of consensus on the evaluation process that should be selected [10].

Past evidence showed that vaccination is among the topics most frequently searched online [11]. Previous content analyses have shown that search engines list approximately as much anti-vaccination as pro-vaccination content [11–13]. However, to date, no evidence exists on differences in quality among pro- and anti-vaccination web contents. If the information disclosed by anti-vaccination web sources is of poor quality, there is a risk that part of the online population is exposed to wrong and hazardous information. Since “consumers may lack the motivation and literacy skills to evaluate information quality of health webpages” [14], the anti-vaccination movement might contribute to increasing unjustified fears, an insufficient vaccination uptake (when it is not a compulsory procedure), and a reemergence of infectious diseases that had almost disappeared in the advanced countries of the world. As a matter of fact, monitoring and assessing the value of online vaccination information appears to be a fundamental step in enhancing the quality of web-based health contents, which might consequently enable individuals to make better health decisions and adopt healthier behaviors.

To date, few content analyses of online vaccination websites have been conducted. As they are often narrowly aimed towards single immunizations (e.g., specifically on HPV vaccination: [15–17]), and as all past assessments on the typology of online vaccination information found a growing amount of anti-vaccination content [11, 18–21], the current study intends to define and compare the quality attributes of pro- and anti-vaccination sources, and in doing so to include multiple types of immunizations.

## Methods

### Source of data (Webpages)

In order to reach our aim, an adequate and comparable number of both pro- and anti-vaccination webpages was needed. Our objective was therefore not to get a representative sample of vaccination-related webpages, but to be able to systematically analyze a large and equal number of pro- and anti-vaccination online sources, to ultimately compare their quality. For this purpose, a pre-determined number of vaccination-related webpages (*N* = 1394) was obtained from two research studies conducted at the Institute of Communication and Health (ICH) at the Università della Svizzera italiana (USI), based in Lugano, Switzerland [22]. These studies had the main objective to investigate user’s knowledge and beliefs toward immunizations after having been exposed to vaccination-related content (i.e., 10 min online session). The independent category was exposure to different combinations of HONcode-certified websites of high quality and webpages with anti-vaccination content. Anti-vaccination sites were retrieved via a customization^1^ of the Google search engine, using keywords such as “vaccination and autism”; “vaccination side effects”; “anti-vaccination movement”. In order to assure traceability of the data set obtained, and ultimately conduct further investigations, the researcher archived the URLs of all the webpages processed in the studies (*N* = 1394). All webpages retrieved for these two studies (i.e., pilot study and experiment) formed the sample for the present content analysis. Webpages were accessed and reviewed between 15 October 2013 and 15 December 2013.

### Exclusion criteria

Webpages were excluded from analyses if they were duplicates (*N* = 19), not written in English (*N* = 0), not anymore available or retrievable (*N* = 40), if they redirected to other web sources (e.g., index pages providing links to articles or news; *N* = 100) or to a URL other than the one originally shown (*N* = 5), if they did not or only marginally treat the topic of vaccination (*N* = 28), had an insufficient amount text to evaluate (*N* = 38), or were delivered via .pdf or similar formats (*N* = 71). Pdf or similar formats were not considered for the present study due to their static nature. This amounted to 300 discarded webpages, which left 1093 webpages for analysis. The exclusion rate of 21.5 % was comparable to those of past studies of the field [23–25].

### Coding instruments

In light of the lack of content analyses focused on quality in the past, a new tool was developed specifically for the purpose of the present study. The tool consisted of three related coding instruments: 1) Online Vaccination Information Quality (OVIQ) codebook; 2) OVIQ code-sheet; and 3) OVIQ checklist. The checklist was designed to simplify raters’ coding efforts by providing a clear and comprehensive graphical view of the entire set of quality indicators and relative values. However, especially in the initial phase of the webpage evaluation, it was highly important that coders understand properly the coding rules stated only in the codebook. The final version of the quality assessment instrument included 40 categories, mostly having a dichotomous value (i.e.*, 0 = not available/ not stated/ not detected, and 1 = available/ stated/ detected*). The other categories either were of qualitative nature (e.g., *Ease of Use, Functioning of Links*) or again quantitative, but with a further degree of specification (e.g., *Type of Information, Bar Menu*).

### Information quality categories

The information quality categories included in the coding scheme were derived from relevant literature pertaining to general health information quality and from research conducted on vaccination information [3, 5, 7, 10, 12–14, 19, 21, 23, 26–48]. Additionally, guidelines developed in the context of several online health information quality initiatives were retrieved through the support of the academic article written by Wilson [8], and considered for the present investigation (The Health On the Net Foundation, HONcode) [49]; URAC^2^; Netscoring^3^; eHealth Code of Ethics^4^; Web Medica Acreditata [50]; and Standford Persuasive Tech Lab [51]).

**Fig. 1:**
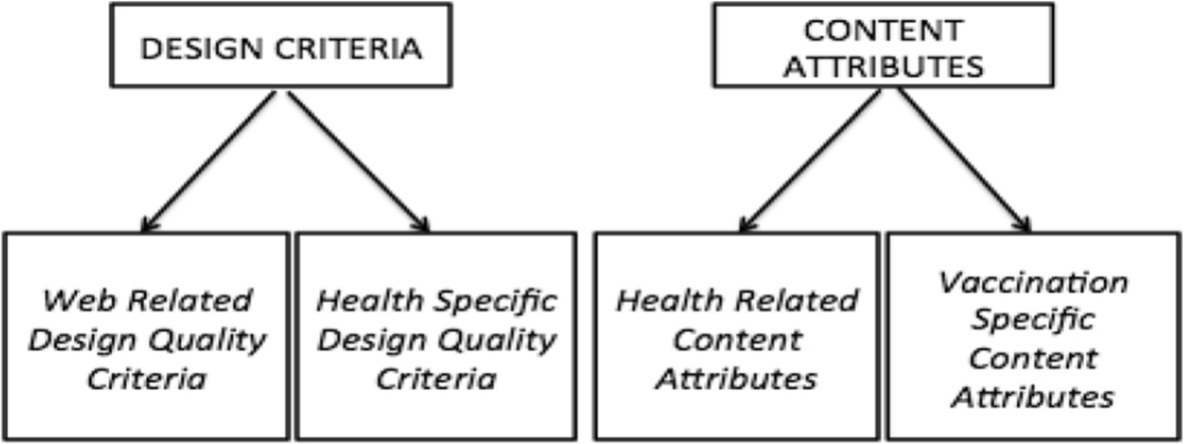
Systems and sub-sets of categories

All relevant categories were segmented into *design* and *content* attributes. *Design quality attributes* incorporated both criteria considered as fundamental when analyzing webpages in general, irrespective of their subject (i.e., *web-related design quality criteria,* first 10 categories), and criteria that are more indicative of the quality status of online health resources (3 categories). *Content quality attributes* were subsequently divided into health-related content attributes pertaining to general health information (12 categories), and vaccination-specific content attributes (15 categories). Figure 1 provides a visual representation of the systems and subsets of categories developed for this study.

After redundant categories were discarded, the final codebook included 40 categories as listed in (see Additional file 1: Table S1).

### Raters and reliability

Two coders carried out the coding process: the first author of the study (GS) and an undergraduate communication scholar familiar with content analysis and trained in applying the coding system (two training sessions of about 2 h each). In a pilot test phase both raters independently applied the codebook to 20 webpages randomly selected from the full sample. This phase was completed without any major glitches (except the need to better specify a few coding rules), and the majority of categories were considered as comprehensible and easily applicable for both raters. A formal reliability assessment phase was then conducted. As results were satisfactory, the undergraduate rater was employed to evaluate additional 150 vaccination-related webpages of the initial sample (*N* = 1394).

The reliability index applied was Cohen’s Kappa [52] because it is conservative and accounts for chance agreement [53], and because all the relevant categories of the coding instrument had a nominal status. Implementing the recommendations provided by Lombard and colleagues [53], the minimum acceptable level of agreement was set at .60.

For testing inter-coder reliability, 100 webpages were randomly selected from the initial sample of 1394 web-links.

As displayed in Additional file 1: Table S1, almost all categories had moderate to perfect agreement levels. Among the quality attributes pertaining to the design macro section, the specific category *Ease of Use (navigability)* had to be excluded from the computation of indices due to its low level of agreement (*k* = .45). Also excluded were the target audience sub-option *caregivers* (*k* = .57) from the specific section health-related content attributes, and the category *risk of not getting vaccinated* (*k* = .33) from the vaccination-specific section. The entire coding instrument (OVIQC) had a high level of agreement (*k* = .89).

### Data processing and analysis

The independent category of this study was the *general tone* of the webpage, which could be pro-vaccination, anti-vaccination, or neutral. General tone was measured as a global assessment of the website’s position in the vaccination controversy. The major dependent category was the presence or absence of the different quality indicators. Categories created to highlight content features that are peculiar either to pro-vaccination online pages only or exclusively for the opposite anti-vaccination contents are not used for comparison purposes. For instance the category labeled as *how to get vaccination exemptions legally* was clearly designed to evaluate contents opposed to the vaccination practice.

The following indices were computed:

1. Webpage Design Index was computed from three of the ten original Web-related design quality criteria, plus the three from the Health-specific design quality criteria, and ranged from 0 to 6.
2. Interactivity Index was computed from two of the ten original Web-related design quality criteria, and ranged from 0 to 6.
3. Health-Related Content Quality Index was computed from eleven of the twelve original Health-related content attributes, plus two from the original Web-related design quality criteria, and ranged from 0 to 13.
4. Vaccination-Specific Content Index was computed from five of the fifteen original Vaccination-specific content attributes, and ranged from 0 to 9.

Table 1 shows the four different indices along with the categories that make them up. The four indices were summed up to form a *Total Aggregated Quality Index*, ranging from 0 to 34. Based on this index, webpages were classified as poor quality (<15 point), medium quality (15–22 points), and high quality (>22 points).

**Table 1:** Online vaccination information quality indices

Webpage design	Interactivity	Health-related content	Vaccination-specific content
• Functioning of links (first 3)^a^ • Bar menu^a^ • Ease of Use (navigability) ^ab^ • Search toolbar • Images • Videos • Graphs/tables/diagrams^a^	• Interactivity presence and requirements^a^ • Interactivity type a) E-mail/newsletter b) Chat rooms c) Forum d) Post comments e) Other interactive services or tools (e.g., share on FB)	• Presence of title • Medical ownership • Target audience^a^ • Date of creation • Date of last update • References of original contents^a^ • Contacts and feedback mechanism^a^ • Accreditations^a^ • Advertising presence^a^ • Complementarity statement • Readability (Flesch-Kincaid Grade Level)^a^ • Language/s • Privacy Policy	• Disease information • Treatment information • Benefits and risks of vaccination a) Benefits of vaccination b) Low risks of vaccination c) Severe risks of vaccination • Definition of terms or (Q&A) • Links to other vaccination related websites a) Links to pro-vaccination websites b) Links to Anti-vaccination websites c) Links to other online health information resources

^a^Quality categories holding values that need to be recoded along with the dichotomous values *0 = not* stated/ not avaliable/ not detected/poor; and 1 = stated/ available/ detected/ valuable

^b^Discarded category after inter-coder reliability tests

Data analysis included frequency counts, cross tabulations, Pearson’s chi-square, and other inferential indicators.

## Results

### Descriptive analysis

#### General tone of the webpage

Of the final sample of 1093 webpages, 514 were pro-vaccination (47 %), 471 were anti-vaccination (43 %), and the residual 108 were neutral or with an undefined tone toward the vaccination practice (10 %). The majority of anti-vaccination pages had radical and opposing viewpoints towards vaccinations (*N* = 430), whereas a minor part adopted a more moderate view on specific immunizations (*anti-vaccination: reformist* 5; *N* = 41). Due to the small number of moderate sites, the two anti-vaccination categories were collapsed in the comparative analysis. It has to be noted that the more or less even split between pro- and anti-vaccination webpages is a consequence of our sampling strategy and is functional at easing the comparison of the two subsets for the present analysis, but should not be taken as an indication about the prevalence of pro- and anti-vaccination views on the web.

#### Non-quality attributes of Pro- and anti-vaccination online information

Pro-vaccination webpages were mainly holding the following Domain Name Systems: .gov (46.7 %), .com (27.6 %), and .org (13.4 %). Strictly linked to this previous finding, half of the webpages were owned by governmental, public, and international institutions (54.1 %), followed by commercial (22.2 %), and not-for-profit organizations (13.4 %). On the other hand, anti-vaccination web sources were mainly published via the following DNSs: .com (48.8 %), .org (33.1 %), and .to (12.3 %). Slightly more than half of the anti-vaccination pages were owned by not-for-profit organizations (56.3 %), and by private individuals (14.4 %). For a consistent portion of anti-vaccination web sources the ownership type of the website was not mentioned clearly (21.7 %). Almost all the pro-vaccination webpages were inserted into general health information web portals (94 %), followed by general vaccine information websites (5.3 %), and the remaining 3 webpages were promoted via specific vaccine websites (0.6 %). Anti-vaccination webpages were mainly published either via general vaccine information websites (47.6 %) or into general health information web portals (49.7 %), and, for the remaining 13 webpages; the raters were not able to detect the scope of the websites that were including them.

For both subsets of the sample, the majority of the contents published were intended for lay people (patients or informal caregivers; 83.9 % of pro- vs. 98.9 of anti-vaccination webpages). However, roughly 30 % of the pro-vaccination pages delivered contents for healthcare professionals (29.2 %), whereas only 6 anti-vaccination pages delivered contents for this audience (1.6 %).

Approximately, 77 % of pro- and 80 % of anti-vaccination web sources offered information about more than a single injection. Barely 7 % of the pages in favor of vaccination provided contents about alternative or natural medicine (or treatments). On the other hand, slightly less than one fourth of the anti-vaccination webpages published those specific contents (23.6 %; less frequent than [19, 41]). Just about 15 % of pro- and only 5 % of anti-vaccination web sources exposed the target audience’s Vaccination Recommendation Schedule (VRS). Out of the 514 webpages being in favor to vaccines, as expected, only three explained to users how to get vaccination exemptions legally (0.6 %). This information was present on slightly less than one-fifth of the anti-vaccination webpages (18.4 %; lower frequency than [19, 41]). Around 14 % of pro-vaccination pages advanced the theme of parents’ rights and amplified responsibilities, especially when they decide not to get vaccinated their children. On the contrary, 37 % of anti-vaccination sources claimed the fact that parents’ (or patients’) rights are violated due to the strict health policies emanated by government bodies with public health executives (less frequent than [19, 41]). 43 % of the web sources opposed to the vaccination practice (lower frequency than [41]), and only 4 % of pro-vaccination pages advanced the fact that potential conflict of interests between health professionals (e.g., physicians) and the pharmaceutical industry might be present. Both pro- and anti-vaccination webpages frequently provided contents about other immunization-related topics (e.g., vaccination information for travelers, health policy change; 56.2 % vs. 58.2 % respectively). The frequency scores for all evaluated attributes fulfilled by pro- and anti-vaccination webpages can be consulted in (see Additional file 2: Table S3).

### Comparative analysis: the quality of pro- vs. Anti-vaccination pages

#### Webpage design index

Webpages holding a favorable view of the vaccination practice showed on average more webpage design features compared to the opponent and neutral vaccination-related sources. There was a statistically significant difference (*p* < .01) when comparing the scores on the aggregated Webpage Design Index of the three groups based on the webpage’s *general tone* [F(2.1090) = 15.35, *p* < 0.001]. Post hoc comparison using the Tukey HSD test showed that the mean Webpage Design Index score for pro-vaccination webpages (M = 3.65, SD = 0.87) was significantly higher than both the anti-vaccination pages (M = 3.27, SD = 1.36, *p* < 0.001), and the neutral (or undefined) set of webpages (M = 3.29, SD = 1.09, *p* = 0.007).

In more detail, pro-vaccination webpages offered better *functioning links* (*p* = 0.01)*,* more often a *bar menu* (*p* < 0.001), or a *search toolbar* (*p* < 0.001). Indeed, 13 out of 15 webpages coded as having from medium to poor quality of links had an anti-vaccination tone. Roughly, one quarter of all the anti-vaccination and neutral webpages did not provide the menu bar feature, and 40 % of the total anti-vaccination subset did not have a user-friendly search toolbar (i.e., 19 % of the neutral webpages neither had this design feature). Images were present on about 60 % of both pro- and anti-vaccination online sources. Many pro- and anti-vaccination pages exposed pictures of people (42 % vs. 35 % respectively), followed by other kind of visuals (e.g., books; 28 % vs. 31.6 % respectively), and about one-fifth of both subsets showed images of drugs and medical equipment (18.7 % vs. 20.6 % respectively). Only about 12 % of pro- and around 8 % of anti-vaccination web sources provided information-supporting formats, such as graphs, tables or diagrams. Remarkably, the anti-vaccination subset provided more video formats than anti-vaccination webpages (27.6 % vs. 10.3 % respectively).

*Videos*, however, can be found most often on anti-vaccination webpages, *p* < 0.001. Almost 28 % of the webpages opposing vaccination included a video format, while only 10 % of pro-vaccination webpages incorporated video formats.

#### Interactivity index

Websites holding a favorable or neutral view toward the vaccination practice provide more interactive services and tools compared to the opponent sources. A statistically significant difference (*p* < .05) was observed when comparing the three groups based on the webpage’s *general tone* on the aggregated Interactivity Index [F(2.1090) = 4.43, *p* = 0.01]. Post hoc comparison using the Tukey HSD test showed that the mean Interactivity Index score for both pro-vaccination webpages (M = 3.71, SD = 0.96, *p* = 0.047), and the neutral (or undefined) subset (M = 3.86, SD = 1.74, *p* = 0.040) was significantly higher than the webpages which were against the vaccination practice (M = 3.49, SD = 1.70). None of the webpages evaluated fulfilled all the 6 categories grouped in this specific index. More specifically, 15 % of the anti-vaccination webpages did not provide any of the *interactive tools or services* coded for the present study while only 3 % of the pro-vaccination subset did not have interactivity, *p* < 0.001. *E-mail or newsletter* services were lacking in 17 % of the anti-vaccination webpages, and on 16 % of the neutral ones: *p* < 0.001. Interestingly, almost one quarter of the neutral web sources reviewed included *forum* interactions, whereas both anti- and pro-vaccination pages offered forums in only 6.6 and 4.7 % of the cases, respectively (*p* < 0.001). A chance to *post comments,* however, was more often offered on neutral (37 %) and anti-vaccination sites (31.8 %) than on the webpages of the pro-vaccination subset (11,5 %) (*p* < 0.001).

#### Health related content index

Webpages holding a favorable view of the vaccination practice endorse more health information quality principles compared to the opponent and neutral vaccination-related online sources. The three groups based on the webpage’s *general tone* presented significant differences (*p* < .01) on the aggregated Health Related Content Index mean score [F(2.1090) = 244.21, *p* < 0.001]. Post hoc comparison using the Tukey HSD test showed that the mean Health Related Content Index score for pro-vaccination webpages (M = 8.00, SD = 1.78) was significantly higher than both the neutral (or undefined) set of webpages (M = 6.32, SD = 1.77, *p* < 0.001), and the anti-vaccination links (M = 5.62, SD = 1.60, *p* < 0.001). Here the maximum score was obtained by the following pro-vaccination webpage: http://www.familydoctor.org/familydoctor/en/kids/vaccines/polio-vaccine.html, which fulfilled all the 13 content quality attributes included in the Health Related Content Index.

The specific categories which played an important role generating this significant effect were: *medical ownership* 6 –80 % of the pro-vaccination webpages were affiliated with a medical organization, compared to only 5.7 % of anti-vaccination pages, and 30 % of the neutral subset – (*p* < 0.001); *target audience* – one quarter of all the pro-vaccination webpages explicitly stated their intended target audience compared to only 9 % of the anti-vaccination, and 11 % of the neutral subset – (*p* < 0.001); *date of last update* –available on half of the pro-vaccination subset, compared to only 12 % of the anti-vaccination and 13 % of the neutral webpages – (*p* < 0.001); *accreditations* – 100 out of 109 accredited webpages evaluated had a favorable view toward the vaccination practice – (*p* < 0.001); *advertising presence* – out of the total number of 114 webpages displaying unclear distinction between core contents and advertising, 107 were anti-vaccination – (*p* < 0.001); *privacy policy* – roughly 90 % of the pro-vaccination webpages properly stated the relative policies about the treatment of confidential data submitted by end-users, compared to only 60 and 77 % reported by the anti-vaccination and the neutral (or undefined) sets of the sample, respectively – (*p* < 0.001); and *complementarity statement* – available on 80 % of the pro-vaccination subset compared to only 56 % of the anti-vaccination, and 40 % of the neutral webpages – (*p* < 0.001). The exception is the *date of creation*, which was indicated more often on both neutral (or undefined) and anti-vaccination webpages (60, and 45 %, respectively) than on the pro-vaccination subset (32 %), (*p* < 0.001). Scientific references of the original contents disclosed were lacking on around 38 % of the pro- and on almost half of the anti-vaccination pages (49 %). Only 27 pro-vaccination pages did not offer contacts information about the owners of the website (5.3 %), while this information was lacking on roughly 17 % of the anti-vaccination subset, resulting impossible for users to contact the responsible person. Only 2 % of pro- and around 3 % of anti-vaccination web sources delivered ‘easy-to-read’ contents (i.e., readability score below or equal the 6^th^ grade levels). One-fifth of both pro- and anti-vaccination webpages showed medium levels of readability (19.1 % vs. 21 % respectively). Indeed the current study showed that the majority of the pages promoted vaccine-related contents that were proved to be difficult to read (i.e., readability score equal or above the 10^th^ grade levels; 79.2 % of pro- vs. 76.2 % of anti-vaccination sources). The provision of another language other than English was adopted by nearly 46 % of pro- and 38 % of anti-vaccination webpages.

**Fig. 2:**
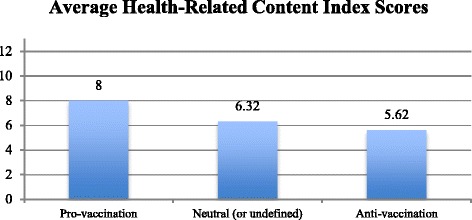
Average health-related content index scores obtained by the three different sub-sets of the sample

Only 180 webpages were judged to target *healthcare professionals*. The majority of them (83.3 %) held a favorable general tone toward immunizations. Neutral (or undefined) and anti-vaccination web sources accounted for 13.3 and 3.4 % of webpages targeted to healthcare professionals respectively. Figure 2 provides a visual representation of the mean Health-Related Content index scores obtained by the three different subsets of the sample.

#### Vaccination-specific content index

Web sites holding a favorable view toward the vaccination practice provide more vaccination-specific information themes compared to the neutral vaccination-related online sources. The anti-vaccination subset, however, has proved to include vaccination-specific information and services features as much as the other two subsets without significant differences. Comparing the scores on the aggregated Vaccination Specific Content Index of the three groups based on the webpage’s general tone, a statistically significant difference (*p* < .05) was observed [F(2.1090) = 3.59, *p* = 0.028]. Post hoc comparison using the Tukey HSD test showed that the mean Vaccination Specific Content Index score for pro-vaccination webpages (M = 5.95, SD = 2.06, *p* = 0.02) was significantly higher than the webpages that were neutral (or undefined) toward the vaccination practice (M = 5.37, SD = 2.60). Remarkably, the maximum score achievable and reported of fulfilling all the 9 vaccination-specific categories was attained by the anti-vaccination subset of webpages (e.g., http://www.prisonplanet.com/myth-busted-vaccinations-are-not-immunizations.html). In detail, neutral webpages conveyed less *disease information* (40 %) than both pro- and anti-vaccination subsets (58 and 52 %, respectively), *p* = 0.001. *Treatment information* was delivered more by the pro-vaccination subset (83 %), compared to both anti-vaccination and neutral (or undefined) webpages (64 and 63 %, respectively), *p* < 0.001. *Benefits, and risks of vaccination* were present in both pro-, and anti-vaccination webpages (79 and 83 %, respectively), but not as much for the subset being neutral (or undefined) in regard to vaccination (56 %), *p* < 0.001. As expected, *benefits of vaccination* were persistently treated by pro-vaccination webpages (75.3 % of the total sample’s subset). About 30 % of neutral, and 10 % of anti-vaccination webpages delivered the benefits derived by immunizations, *p* < 0.001. Conversely, *severe risks of vaccination* were insistently promoted by anti-vaccination webpages (78 % of the total subset of the sample), followed by the neutral (or undefined) subset (50 %). One fifth of pro-vaccination webpages advanced the sub-topic (or theme) of the serious complications potentially caused by several immunizations, *p* < 0.001. The provision of *terms’ definitions and Q&A formats* was significantly less frequent in anti-vaccination webpages (only 16 %), as compared to both pro-vaccination (29 %) and neutral subsets (35 %), *p* < 0.001. The inclusion of external *links to other vaccination-related web sources* was, as estimated, a central component for all the subsets of webpages investigated (around 85 % to 90 % each subset). In particular, a positive relationship was observed between a favorable tone toward the vaccination practice and the provision of external links to *other pro-vaccination sources* (77 %), *p* < 0.001. About half of neutral (or undefined), and one quarter of anti-vaccination webpages reviewed were externally linked with further pro-vaccination websites. On the other hand, external *links to* supplementary *anti-vaccination websites* were positively correlated with the negative tone of the webpage (83 %), *p* < 0.001. About 60 % of neutral (or undefined), and only 4 % of pro-vaccination webpages offered links to external anti-vaccination websites.

#### Total aggregated quality index

Webpages holding a positive tone toward the vaccination practice fulfilled consistently more quality criteria included in the present content analysis when compared to the opponent and neutral vaccination related online sources. It has however to be noted that none of the three different subsets obtained average scores falling into the high quality range (>22 points). The analysis revealed significant differences (*p* < .01) when comparing the scores on the Total Aggregated Quality Index of the three groups based on the webpage’s general tone [F(2.1090) = 66.49, *p* < 0.001]. Post hoc comparison using the Tukey HSD test showed that the mean Total Aggregated Quality Index score for pro-vaccination webpages (M = 21.31, SD = 3.64) was significantly higher than both the anti-vaccination links (M = 18.25, SD = 4.57, *p* < 0.001), and the neutral (or undefined) set of webpages (M = 18.84, SD = 5.30, *p* < 0.001). By taking a deeper look at these findings, it has to be observed that none of the webpages included in the final sample of the present study comprehensively fulfilled all the 34 information quality categories coded. The maximum score reported was 31 categories fulfilled by the following pro-vaccination webpage: http://www.netdoctor.co.uk/conditions/pregnancy-and-family/a9117/childhood-vaccinations/.

**Fig. 3:**
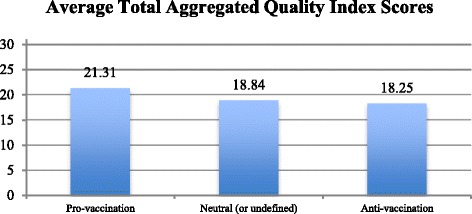
Average total aggregated quality index scores obtained by the three sub sets of webpages

Figure 3 offers a graphical representation of the mean Total Aggregated Quality Index scores acquired by the three subsets of webpages.

## Discussion

The main aim of this study was to compare the quality of pro- and anti-vaccination webpages. Our analyses highlighted significant differences between pro-vaccination, anti-vaccination, and neutral (or undefined) webpages along all four quality dimensions: webpage design, interactivity, health-related content, and vaccination-specific content. Generally speaking, pro-vaccination webpages resulted to be qualitatively superior to both anti-vaccination and neutral (or undefined) web sources (Total Aggregated Quality Index). However, on some quality features, neutral and/or anti-vaccination webpages showed better results than the pro-vaccination subset.

According to our analyses, higher quality of pro-vaccination websites might be explained by higher professionalism of their owners, who were more often internationally recognized medical institutions (e.g., WHO, CDC), while anti-vaccination websites were often operated by activists of the anti-vaccination movement, that is private citizens expressing their personal views on the topic. This difference makes it likely that anti-vaccination websites are less often designed professionally and may have difficulties to keep up to date with regard to quality standards. However, it has to be noted that various counterexamples were observed with regard to the quality levels of some anti-vaccination webpages. Eighty-seven anti-vaccination webpages reviewed obtained high quality scores, satisfying between 23 and 29 of the quality attributes. For instance, the following anti-vaccination webpage: http://articles.mercola.com/sites/articles/archive/2011/11/01/more-parents-waking-up-to-vaccine-dangers.aspx obtained a total aggregated quality score of 29 attributes out of 34. The website is administered by a well-known American doctor and activist (Dr. Joseph Mercola), and it promotes a wide range of health information such as wellness, dietary, and vaccines. The website has been ranked by Alexa.com the ‘World’s first Natural Health Website’. A further well-known counterexample is represented by the National Vaccine Information Center (NVIC), which is a non-profit organization advocating mainly vaccine safety (http://www.nvic.org/).

Past research [39] has claimed that the most reliable domain name systems to retrieve quality health information might be the following three: .gov, .org, and .edu. However, given that the .org domain was also frequently the DNS of anti-vaccination web sources in our sample, we suggest that in the context of online vaccination information this should be limited only to the .gov and .edu domain options. In light of this, international and national bodies monitoring online vaccination information have to stress the positive relationship between governmental institutions (i.e., holding the .gov domain) and the provision of quality contents related to vaccination. For end-users, the identification of the DNS might be a simple and quick way to infer the credibility of a webpage disclosing information on immunizations (governmental or public affiliation).

With respect to aspects relating to webpage design, pro-vaccination web information was disclosed through well-organized web portals with highly operative internal links (i.e., increasing application of menu bars, and search toolbars), and, as a matter of fact, have to be considered aesthetically more pleasant to navigate than anti-vaccination webpages (i.e., lack of distracting promotional messages). Remarkably, the adoption of “two-way communication” formats resulted to be high (90.6 % of the final sample), and on average webpages being pro-vaccination satisfied this aspect better than the opponent subset. This finding may highlight the difficulties of web editors administering anti-vaccination webpages in designing modern and dynamic online platforms, which force end-users to consume contents only in a static traditional fashion.

The most notable exception to the quality gap between pro- and anti-vaccination websites occurred for the vaccination-specific information section. In this dimension, pro- and anti-vaccination websites were found to be equally informative. But does this mean the information on anti-vaccination websites is as good as on pro-vaccination-sites? Not necessarily so. The indicators employed in this study used the presence of information as criterion, not its relevance, not its substance, not its truthfulness. These are the ultimate quality criteria, nevertheless they are very difficult to measure and next to impossible to assess on a large-scale quantitative basis. This is why our study and its forebears use proxies and shortcuts aiming at the presence of information. We have to keep in mind that what is present need not necessarily be helpful or truthful.

Consistently with previous research on health information quality (e.g., [7, 14, 23]), the findings of the present study confirmed the average quality level of pro- and anti-vaccination information available online. This result could be taken as an indication that not much progress was made with regard to the quality of health related websites since the earlier studies were fielded. Moreover, according to the readability assessment tool applied (Flesch-Kincaid Grade Level), the majority of webpages reviewed were written in a demanding manner. Indeed, webpages’ readability levels were higher than the average American reading level, which is set between 7^th^ and 9^th^ grade (see: [36]). This latter negative result, which is coherent with past research evaluating online health information (e.g., [7, 45]), emphasizes the potential shortcomings of the Web as a complementary source of health information, and especially as a medium to retrieve and exchange vaccination-related information. As advanced by Fagerlin, Wang and Ubel [54], given the complex nature of health information, websites promoting vaccination have to carefully design their messages in a way that are in line not only with the target audience’s needs and “prior knowledge but also with their capacity to process the information, such as numeracy and health literacy, as well as their preferences for how information is presented” (as cited in [12], p. 3731).

### Implications for online health information providers

The findings of this study, based on a large sample size (*N* =1’093), allow us to draw several conclusions that can be translated into advice to online health information providers on how to make vaccination-related webpages more accurate, complete, attractive, and easy-to-navigate.

First, it is evident that to be supportive, complementary, and beneficial for everyone, health information available on the Internet has to be written below the average reading level (between 7^th^ and 9^th^ grade level) (e.g., [7, 36]). The increasing availability of online readability assessment tools (e.g., http://www.readability-score.com/) opens the door to web editors to easily implement this first and relevant suggestion. Another way to assure understandability of the information provided might be the adoption of “Question and Answer” (or FAQ) formats.

Second, given that the frequency of inclusion of pictures related to vaccination preventable diseases’ (VPDs) effects resulted to be low, future educational vaccination-related web sources, in order to stress the perceived severity of VPDs, might include more often visual representations. Another feature, which is now missing across webpages, and most likely represents a useful aid to consumers seeking online vaccination information, is the target audience’s “vaccination recommendation schedule”. Additionally, the large number of videos available on anti-vaccination websites might be balanced by pro-vaccination pages providing more visual recordings that enable users to understand in a vivid manner the positive effects of vaccinations, and the risk derived by contracting a vaccine-preventable disease (e.g., showing, in form of narrative, a sad mother which lost her son due to a VPD) [12]. Moreover, in order to facilitate understandability of the contents provided, the application of videos/animations is especially suited for low health literate individuals [55]. Third, the so-called “*post comment* on this page” feature is still missing on a large portion of webpages being in favor to immunizations. This type of interactive service has the potential to increase users’ involvement, and, consequently, the relative effect of messages on them [56, 57] as well as attention, knowledge, perception of being socially supported, and ultimately positive health outcomes [58, 59].

Fourth, since secondary ethnic groups are present in many English-speaking countries, vaccination web sources might offer the selection of a further language (e.g., Spanish).

### Limitations and further research

The present study has some limitations. First, as the nature of Internet information is purely dynamic (updating processes), the currency of the results gained from this content analysis may have been already challenged. Secondly, while some of the webpages were retrieved with the original Google search engine, the rest of them were obtained through two manipulated versions of the same engine. In particular, one engine was filtered (with a set of predefined keywords) to only search for vaccination-related links holding a negative tone, and the other to yield only pages from reputable sources (mixes of HONcode certified sites). Thus, some of the webpages reviewed might not have been retrieved by typical online end-users. However, the applied keyword strategy considered a set of numerous terms, which could have been easily used by consumers on a normal online information seeking session. The initial sample represented also another constraint for the results of the current study. Pro-vaccination webpages were obtained through the application of a filter manipulating the original Google search engine to yield sources of online health information certified by the Health On the Net Foundation (HON) and other highly credible online sources. This aspect has directly influenced our descriptive, and with minor impact, our comparative findings that have to be interpreted with caution*.* Last, it has to be noted that the final sample of 1093 web links included various webpages owned by the same website (e.g., www.cdc.gov webpages were around 100).

The findings of the present study can be extended to future comparative analyses in other fields^7^. In fact other qualitative categories apart from the core one (“*general tone”* of the webpage) can be employed in the same way as the principal construct. For instance, mean scores of the four aggregated indices can be calculated, among others, for the category labeled as “*scope of information of the website*”, or again for the “*domain name system*” (DNS) construct.

Our content analysis did not code the specific type of vaccination (e.g., HPV) or related disease (e.g., mumps) presented by the different webpages reviewed. We recommend future studies evaluating vaccination-related web sources to include this data to ultimately conduct more sophisticated comparative analyses. Future studies are also necessary to build solid findings that both test the instrument’s usability, and reconfirm the strong inter-rater agreement (*k* = .89) attributed to the present assessment tool.

## Conclusions

An evaluating instrument has been designed for assessing the quality of vaccination webpages. Pro-vaccination webpages on average yielded better results than the anti-vaccination subset in term of design, interactivity, and health information quality, but not on the specific degree of inclusion of common and relevant vaccination themes. This content analysis alarmingly showed that reading levels of online vaccination information are moderately high, thus disrespecting the recommended viable levels emanated by numerous public health organizations. This negative and significant trend confirms, perhaps, one of the most evident shortcomings attributed to the “hyperlinked” World Wide Web when accessing health information.

## Additional files

Additional file 1: Table S1. Comparing the Quality of Pro- and Anti-Vaccination Online Information: A Content Analysis of Vaccination-Related Webpages.pdf. (DOCX 83 kb)

Additional file 2: Table S3. Comparing the Quality of Pro- and Anti-Vaccination Online Information: A Content Analysis of Vaccination-Related Webpages.pdf. (DOCX 29 kb)

## Abbreviations

HON: health On the net foundation
OVIQC: Online Vaccination Information Quality Codebook
OVIQ: Online Vaccination Information Quality

## Footnotes

1 “The customization of the search engines was done by limiting their search coverage to different pre-defined sets of websites […]. This manipulation was realized by configuring the context and annotation files of Google custom search engine” [22]. Google custom search engine is available at: https://developers.google.com/custom-search/

2 American Accreditation HealthCare Commission: https://www.urac.org/

3 NetScoring quality criteria: http://www.chu-rouen.fr/netscoring/netscoringeng.html

4 Internet Healthcare Coalition: http://www.ihealthcoalition.org/ehealth-code-of-ethics/

5 As explained by Hobson-West [43], the anti-vaccination group labeled as reformist is composed by those people “who are critical of vaccines but likely to provide at least partial support to vaccination”.

6 For the current study the category *medical ownership* was specifically designed to depict if on the page evaluated the name of a doctor or a medical corporation was present. However, it has to be noted that no disctinctions were made in case the webpage was affiliated with a single medical doctor or an internationally well-known health institution.

7 The developed evaluating tool (OVIQC) can be obtained by e-mail at the following address (gabriele.sak@usi.ch).
