# Supplementary material for: Comparing the quality of pro- and anti-vaccination online information: a content analysis of vaccination-related webpages
Source: BMC Public Health. 2016 Jan 15;16:38. doi: 10.1186/s12889-016-2722-9 (PMC4714533; doi:10.1186/s12889-016-2722-9)
Supplement: Supplementary file 1 — Comparing the Quality of Pro- and Anti-Vaccination Online Information: A Content Analysis of Vaccination-Related Webpages.pdf. (DOCX 83 kb) [file 12889_2016_2722_MOESM1_ESM.docx]

*Table S1: Overview of Categories – Inter-coder Reliability, Results and Significance Testing*

|  | *Reliability* | *Results: presence of quality indicators* | | | | *Significance test* | | | |
| --- | --- | --- | --- | --- | --- | --- | --- | --- | --- |
| *Variable* | Cohen’s Kappa | *Pro-vaccination N = 514 (47%)* | *Neutral (or undefined) N = 108 (9.9%)* | *Anti- vaccination*  *N = 471 (43.1%)* | *All web pages*  *N = 1093 (100%)* | *Chi squared/F* | | *df* | *p* |
| *1) DESIGN ATTRIBUTES* | .93 |  |  |  |  |  |  | |  |
| *a) Web-related design quality criteria* | .94 |  |  |  |  |  |  | |  |
| *Domain name of the website* | 1.00 |  |  |  |  | 437.75 | 16 | | < .001 |
| .com |  | 142 (32.9) | 58 (13.5) | 230 (53.6) | 430 (39.4) |  |  | |  |
| .gov |  | 240 (93.8) | 15 (5.9) | 1 (0.4) | 256 (23.4) |  |  | |  |
| .edu |  | 11 (91.7) | 1 (8.3) | - (-) | 12 (1.1) |  |  | |  |
| .org |  | 69 (27.8) | 23 (9.3) | 156 (62.9) | 248 (22.7) |  |  | |  |
| .net |  | 2 (7.1) | 3 (10.7) | 23 (82.1) | 28 (2.6) |  |  | |  |
| .info |  | 1 (50) | - (-) | 1 (50) | 2 (0.2) |  |  | |  |
| .int |  | 24 (92.3) | 1 (3.8) | 1 (3.8) | 26 (2.4) |  |  | |  |
| .to |  | 2 (3.1) | 5 (7.7) | 58 (89.2) | 65 (5.9) |  |  | |  |
| Other (DNS) (e.g., .co.uk, etc.) |  | 23 (88.5) | 2 (7.7) | 1 (3.8) | 26 (2.4) |  |  | |  |
|  |  |  |  |  |  |  |  | |  |
| *Functioning of links* | .80 |  |  |  |  | 16.73 | 6 | | .01 |
| No links available |  | 2 (20) | 2 (20) | 6 (60) | 10 (0.9) |  |  | |  |
| High quality of links |  | 511 (47.8) | 105 (9.8) | 452 (42.4) | 1068 (97.7) |  |  | |  |
| Medium quality of links |  | 1 (12.5) | 1 (12.5) | 6 (75) | 8 (0.7) |  |  | |  |
| Poor quality of links |  | - (-) | - (-) | 7 (100) | 7 (0.6) |  |  | |  |
|  |  |  |  |  |  |  |  | |  |
| *Language/s* | .95 |  |  |  |  | 28.65 | 2 | | < .001 |
| Only English available |  | 279 (42.3) | 88 (13.3) | 292 (44.4) | 559 (60.3) |  |  | |  |
| Other languages available |  | 235 (54.1) | 20 (4.6) | 179 (41.2) | 434 (39.7) |  |  | |  |
|  |  |  |  |  |  |  |  | |  |
| *Bar menu* | .91 |  |  |  |  | 206.92 | 4 | | < .001 |
| Not available |  | 70 (33.3) | 27 (12.9) | 112 (53.8) | 209 (19.2) |  |  | |  |
| Available: one bar menu |  | 242 (36.8) | 70 (10.7) | 345 (52.5) | 657 (60.1) |  |  | |  |
| Available: multiple menu bars |  | 202 (89) | 11 (4.8) | 14 (6.2) | 227 (20.7) |  |  | |  |
| *Search toolbar* | .89 |  |  |  |  | 195.41 | 2 | | < .001 |
| Not available |  | 21 (9.1) | 20 (8.6) | 190 (82.3) | 231 (21.2) |  |  | |  |
| Available |  | 493 (57.2) | 88 (10.2) | 281 (32.6) | 862 (78.2) |  |  | |  |
|  |  |  |  |  |  |  |  | |  |
| *Ease of Use (navigability) ^a^* | .45 |  |  |  |  | 217,84 | 4 | | < .001 |
| Easy to navigate |  | 460 (59.7) | 84 (10.9) | 225 (29.4) | 769 (70.3) |  |  | |  |
| Medium ease to navigate |  | 53 (19.4) | 23 (8.4) | 197 (72.2) | 273 (25) |  |  | |  |
| Difficult to navigate |  | 1 (2) | 1 (2) | 49 (96.1) | 51 (4.7) |  |  | |  |
|  |  |  |  |  |  |  |  | |  |
| *Interactivity presence and requirements* | 1.00 |  |  |  |  | 44.55 | 2 | | < .001 |
| Not available |  | 17 (16.5) | 12 (11.7) | 74 (71.8) | 103 (9.4) |  |  | |  |
| Available with registration only |  | 497 (50.2) | 96 (9.7) | 397 (40.2) | 990 (90.6) |  |  | |  |
| Available without registration |  |  |  |  | - (-) |  |  | |  |
| *Interactivity type* | 1.00 |  |  |  |  | 44.55 | 2 | | < .001 |
| Not available |  | 17 (16.5) | 12 (11.7) | 74 (71.8) | 103 (9.4) |  |  | |  |
| Available |  | 497 (50.2 | 96 (9.7) | 397 (40.2) | 990 (90.6) |  |  | |  |
|  |  |  |  |  |  |  |  | |  |
| E-mail/newsletter | .93 |  |  |  |  | 35.80 | 2 | | < .001 |
| Not available |  | 27 (21.8) | 17 (13.7) | 80 (64.5) | 124 (11.3) |  |  | |  |
| Available |  | 487 (50.2) | 91 (9.4) | 391 (40.4) | 969 (88.7) |  |  | |  |
|  |  |  |  |  |  |  |  | |  |
| Chat rooms | 1.00 |  |  |  |  | 1.37 | 2 | | .503 |
| Not available |  | 511 (46.9) | 108 (9.9) | 470 (43.2) | 1089 (99.6) |  |  | |  |
| Available |  | 3 (75) | - (-) | 1 (25) | 4 (0.4) |  |  | |  |
|  |  |  |  |  |  |  |  | |  |
| Forum | .84 |  |  |  |  | 58.74 | 2 | | < .001 |
| Not available |  | 490 (48.5) | 80 (7.9) | 440 (43.6) | 1010 (92.4) |  |  | |  |
| Available |  | 24 (28.9) | 28 (33.7) | 31 (37.3) | 83 (7.6) |  |  | |  |
|  |  |  |  |  |  |  |  | |  |
| Post comment | 1.00 |  |  |  |  | 72.40 | 2 | | < .001 |
| Not available |  | 455 (53.9) | 68 (8.1) | 321 (38) | 844 (77.1) |  |  | |  |
| Available |  | 59(23.6) | 40 (16) | 150 (60.4) | 249 (22.9) |  |  | |  |
|  |  |  |  |  |  |  |  | |  |
| Other interactive services and tools | .95 |  |  |  |  | 5.02 | 2 | | .081 |
| Not available |  | 175 (42.7) | 42 (10.2) | 192 (47.1) | 409 (37.5) |  |  | |  |
| Available |  | 339 (49.6) | 66 (9.6) | 279 (40.8) | 684 (62.5) |  |  | |  |
|  |  |  |  |  |  |  |  | |  |
|  |  |  |  |  |  |  |  | |  |
| *Interactive form* | 1.00 |  |  |  |  | 44.55 | 2 | | < .001 |
| Not available |  | 17 (16.5) | 12 (11.7) | 74 (71.8) | 103 (9.4) |  |  | |  |
| Available |  | 497 (50.2) | 96 (9.7) | 397 (40.2) | 990 (90.6) |  |  | |  |
|  |  |  |  |  |  |  |  | |  |
| User-Provider (or Provider-User) | .94 |  |  |  |  | 36.09 | 2 | | < .001 |
| Not available |  | 28 (22) | 17 (13.4) | 82 (64.6) | 127 (11.6) |  |  | |  |
| Available |  | 486 (50.3) | 91 (9.4) | 389 (40.3) | 966 (88.4) |  |  | |  |
|  |  |  |  |  |  |  |  | |  |
| User-User with moderating profile | .85 |  |  |  |  | 3.35 | 2 | | .187 |
| Not available |  | 505 (47.3) | 103 (9.7) | 459 (43) | 1067 (97.5) |  |  | |  |
| Available |  | 9 (33.3) | 5 (18.5) | 12 (48.1) | 26 (2.5) |  |  | |  |
|  |  |  |  |  |  |  |  | |  |
| User-User without moderating profile | .95 |  |  |  |  | 3.75 | 2 | | .153 |
| Not available |  | 204 (44.2) | 43 (9.3) | 214 (46.5) | 461 (42.2) |  |  | |  |
| Available |  | 310 (49.1) | 65 (10.3) | 257 (40.7) | 632 (57.8) |  |  | |  |
| *Privacy Policy* | .94 |  |  |  |  | 109.27 | 2 | | < .001 |
| Not available |  | 59 (21.6) | 24 (8.8) | 189 (69.6) | 272 (25) |  |  | |  |
| Available |  | 455 (55.4) | 84 (10.2) | 282 (34.3) | 821 (75) |  |  | |  |
|  |  |  |  |  |  |  |  | |  |
|  |  |  |  |  |  |  |  | |  |
| b) *Health-specific design quality criteria* | .92 |  |  |  |  |  |  | |  |
| *Images* | .97 |  |  |  |  | .178 | 2 | | .915 |
| Not available |  | 202 (46.4) | 42 (9.7) | 191 (43.9) | 435 (39.8) |  |  | |  |
| Available showing: |  | 312 (47.3) | 66 (10) | 280 (42.6) | 658 (60.2) |  |  | |  |
|  |  |  |  |  |  |  |  | |  |
| Drugs and medical equipment | .97 |  |  |  |  | .798 | 2 | | .671 |
| Not available |  | 418 (47.4) | 89 (10.1) | 374 (42.5) | 881 (80.6) |  |  | |  |
| Available |  | 96 (45.3) | 19 (9) | 97 (45.8) | 212 (19.4) |  |  | |  |
|  |  |  |  |  |  |  |  | |  |
| Vaccination preventable diseases | .70 |  |  |  |  | 18.39 | 2 | | < .001 |
| Not available |  | 492 (46.2) | 103 (9.7) | 470 (44.2) | 1065 (97.4) |  |  | |  |
| Available |  | 22 (78.6) | 5 (17.9) | 1 (3.6) | 289 (2.6) |  |  | |  |
|  |  |  |  |  |  |  |  | |  |
| People | .90 |  |  |  |  | 4.37 | 2 | | .112 |
| Not available |  | 298 (45.4) | 59 (9) | 299 (45.6) | 656 (60) |  |  | |  |
| Available |  | 216 (49.3) | 49 (11.2) | 172 (39.5) | 437 (40) |  |  | |  |
| Other pictures | .83 |  |  |  |  | 4.34 | 2 | | .114 |
| Not available |  | 369 (48.6) | 67 (8.8) | 322 (42.6) | 758 (69.4) |  |  | |  |
| Available |  | 145 (43.3) | 41 (12.2) | 149 (44.5) | 335 (30.6) |  |  | |  |
|  |  |  |  |  |  |  |  | |  |
| *Videos* | 1.00 |  |  |  |  | 60.80 | 2 | | < .001 |
| Not available |  | 461 (51) | 101 (11.2) | 341 (37.8) | 903 (82.6) |  |  | |  |
| Available showing: |  | 53 (27.9) | 7 (3.7) | 130 (68.4) | 190 (17.4) |  |  | |  |
|  |  |  |  |  |  |  |  | |  |
| Caregiving profile | 1.00 |  |  |  |  | 7.68 | 2 | | .022 |
| Not available |  | 505 (47.6) | 106 (10) | 449 (42.4) | 1060 (97) |  |  | |  |
| Available |  | 9 (27.3) | 2 (6.1) | 22 (66.7) | 33 (3) |  |  | |  |
|  |  |  |  |  |  |  |  | |  |
| Patient | .88 |  |  |  |  | 2.30 | 2 | | .316 |
| Not available |  | 498 (47.2) | 106 (10) | 450 (42.7) | 1054 (96.4) |  |  | |  |
| Available |  | 16 (41) | 2 (5.1) | 21 (53.8) | 39 (3.6) |  |  | |  |
|  |  |  |  |  |  |  |  | |  |
| Health professional | 1.00 |  |  |  |  | 3.66 | 2 | | .160 |
| Not available |  | 488 (47.1) | 106 (10.2) | 441 (42.7) | 1035 (94.7) |  |  | |  |
| Available |  | 26 (44.8) | 2 (3.4) | 30 (51.7) | 58 (5.3) |  |  | |  |
|  |  |  |  |  |  |  |  | |  |
| Other kind of records | .89 |  |  |  |  | 28.46 | 2 | | < .001 |
| Not available |  | 478 (48.9) | 104 (10.6) | 394 (40.4) | 976 (89.3) |  |  | |  |
| Available |  | 36 (30.8) | 4 (3.4) | 77 (65.8) | 117 (10.7) |  |  | |  |
|  |  |  |  |  |  |  |  | |  |
| *Graphs/tables/diagrams* | .94 |  |  |  |  | 40.17 | 8 | | < .001 |
| Not available |  | 451 (45.7) | 100 (10.1) | 434 (44.1) | 985 (90.1) |  |  | |  |
| Available: graph |  | 7 (23.3) | - (-) | 23 (76.7) | 30 (2.7) |  |  | |  |
| Available: table |  | 42 (75) | 5 (8.9) | 9 (16.1) | 56 (5.1) |  |  | |  |
| Available: diagram |  | - (-) | - (-) | 1 (100) | 1 (0.1) |  |  | |  |
| Available: a combination of the above |  | 14 (66.7) | 3 (14.3) | 4 (19) | 21 (1.9) |  |  | |  |
| *2) CONTENT ATTRIBUTES*  *a) Health-related content attributes* | .86  .87 |  |  |  |  |  |  | |  |
| *Presence of title* | 1.00 |  |  |  |  | 4.27 | 2 | | .118 |
| Not available |  | 3 (23.1) | 3 (23.1) | 7 (53.8) | 13 (1.2) |  |  | |  |
| Available |  | 511 (47.3) | 105 (9.7) | 464 (43) | 1080 (98.8) |  |  | |  |
| *Ownership type of the web site* | .94 |  |  |  |  | 586.30 | 10 | | < .001 |
| Not able to detect |  | 27 (19.3) | 10 (7.1) | 102 (73.6) | 139 (12.8) |  |  | |  |
| Detected: government or public institution |  | 278 (93.6) | 18 (6.1) | 1 (0.3) | 297 (27.1) |  |  | |  |
| Detected: academic |  | 13 (86.7) | 2 (13.3) | - (-) | 15 (1.4) |  |  | |  |
| Detected: not-for-profit organization |  | 69 (19.2) | 25 (7) | 265 (73.8) | 359 (32.8) |  |  | |  |
| Detected: commercial |  | 114 (67.1) | 21 (12.4) | 35 (20.6) | 170 (15.5) |  |  | |  |
| Detected: individual homepage |  | 13 (11.5) | 32 (28.3) | 68 (60.2) | 113 (10.3) |  |  | |  |
|  |  |  |  |  |  |  |  | |  |
| *Medical Ownership* | .95 |  |  |  |  | 563.50 | 2 | | < .001 |
| Not available |  | 102 (16.4) | 75 (12.1) | 444 (71.5) | 621 (56.9) |  |  | |  |
| Available |  | 412 (87.3) | 33 (7) | 27 (5.7) | 472 (43.1) |  |  | |  |
|  |  |  |  |  |  |  |  | |  |
| *Purpose of the web page* | .66 |  |  |  |  | 7.68 | 4 | | .104 |
| Not able to detect |  | - (-) | - (-) | 1 (100) | 1 (0.1) |  |  | |  |
| Detected: informational or educational |  | 512 (47.4) | 107 (9.9) | 460 (42.7) | 1079 (98.7) |  |  | |  |
| Detected: promotional, commercial |  | 2 (15.4) | 1 (7.7) | 10 (76.9) | 13 (1.2) |  |  | |  |
| *Target audience* | .87 |  |  |  |  | 70.94 | 4 | | < .001 |
| Not explicitly stated and not able to detect |  | 1 (12.5) | 5 (62.5) | 2 (25) | 8 (0.7) |  |  | |  |
| Not explicitly stated but detected: |  | 384 (42.6) | 91 (10.1) | 425 (47.3) | 900 (82.4) |  |  | |  |
| \| Explicitly stated in the head (or title) of the web page: \|  \|  \|  \|  \|  \|  \|  \|  \| \| --- \| --- \| --- \| --- \| --- \| --- \| --- \| --- \| --- \| |  | 129 (69.7) | 12 (6.5) | 44 (23.8) | 85 (16.9) |  |  | |  |
|  |  |  |  |  |  |  |  | |  |
| Professionals | .73 |  |  |  |  | 142.35 | 2 | | < .001 |
| Not available |  | 364 (39.8) | 84 (9.2) | 465 (51) | 913 (83.5) |  |  | |  |
| Available |  | 150 (83.3) | 24 (13.3) | 6 (3.3) | 180 (16.5) |  |  | |  |
|  |  |  |  |  |  |  |  | |  |
| Patients | .69 |  |  |  |  | 1.01 | 2 | | .602 |
| Not available |  | 232 (45.4) | 53 (10.4) | 226 (44.2) | 511 (46.7) |  |  | |  |
| Available |  | 282 (48.4) | 55 (9.4) | 245 (42.2) | 582 (53.3) |  |  | |  |
|  |  |  |  |  |  |  |  | |  |
| Caregivers ^a^ | .57 |  |  |  |  | 22.61 | 2 | | < .001 |
| Not available |  | 197 (56.3) | 38 (10.9) | 114 (32.9) | 349 (32) |  |  | |  |
| Available |  | 397 (42.6) | 70 (9.4) | 357 (48) | 744 (68) |  |  | |  |
|  |  |  |  |  |  |  |  | |  |
| *Date of creation* | .94 |  |  |  |  | 37.34 | 2 | | < .001 |
| Not available |  | 349 (53.8) | 43 (6.6) | 256 (39.6) | 648 (59.3) |  |  | |  |
| Available |  | 165 (37.1) | 65 (14.6) | 215 (48.3) | 445 (40.7) |  |  | |  |
|  |  |  |  |  |  |  |  | |  |
|  |  |  |  |  |  |  |  | |  |
| *Date of last update* | .95 |  |  |  |  | 191.94 | 2 | | < .001 |
| Not available |  | 254 (33.2) | 94 (12.3) | 416 (54.5) | 764 (69.8) |  |  | |  |
| Available |  | 260 (78.2) | 14 (4.2) | 55 (17) | 329 (30.2) |  |  | |  |
|  |  |  |  |  |  |  |  | |  |
| *References of original contents* | .95 |  |  |  |  | 98.93 | 6 | | < .001 |
| Not available |  | 195 (41.3) | 48 (10.2) | 229 (48.5) | 472 (43.1) |  |  | |  |
| Available: in text only |  | 27 (22.5) | 9 (7.5) | 83 (70) | 119 (11) |  |  | |  |
| Available: at the end of the text only (or at the beginning) |  | 239 (63.9) | 42 (11.2) | 93 (24.9) | 374 (34.2) |  |  | |  |
| Both information available |  | 53 (41.4) | 9 (7) | 66 (51.6) | 128 (11.7) |  |  | |  |
|  |  |  |  |  |  |  |  | |  |
| *Contacts and feed mechanisms* | .85 |  |  |  |  | 162.00 | 4 | | < .001 |
| Not available |  | 27 (22.9) | 13 (11) | 78 (66.1) | 118 (10.8) |  |  | |  |
| Available only one contact feature |  | 184 (33.9) | 61 (11.2) | 297 (54.9) | 542 (49.6) |  |  | |  |
| More than one contact information available |  | 303 (70) | 34 (7.9) | 96 (22.2) | 433 (39.6) |  |  | |  |
|  |  |  |  |  |  |  |  | |  |
| *Accreditations* | .88 |  |  |  |  | 112.51 | 6 | | < .001 |
| Not available |  | 414 (42) | 104 (10.6) | 466 (47.4) | 984 (90) |  |  | |  |
| Available: generic information quality seal |  | 2 (28.6) | - (-) | 5 (71.4) | 7 (0.6) |  |  | |  |
| Available: specific health information quality seal |  | 86 (95.6) | 4 (4.4) | - (-) | 90 (8.2) |  |  | |  |
| Both generic and specific information quality seals available |  | 12 (100) | - (-) | - (-) | 12 (1.1) |  |  | |  |
|  |  |  |  |  |  |  |  | |  |
| *Advertising presence* | .91 |  |  |  |  | 154.57 | 4 | | < .001 |
| Not available |  | 364 (56.3) | 53 (8.2) | 229 (35.5) | 646 (59.1) |  |  | |  |
| Available: clear distinction between core contents and advertising |  | 146 (43.8) | 52 (15.6) | 135 (40.5) | 333 (30.4) |  |  | |  |
| Available: unclear distinction between core contents and advertising |  | 4 (3.5) | 3 (2.6) | 107 (93.9) | 114 (10.4) |  |  | |  |
|  |  |  |  |  |  |  |  | |  |
| *Complementarity statements* | .81 |  |  |  |  | 94.75 | 2 | | < .001 |
| Not stated |  | 93 (26.1) | 56 (15.7) | 206 (58.1) | 355 (32.5) |  |  | |  |
| Stated |  | 421 (57) | 52 (7) | 265 (35.9) | 738 (67.5) |  |  | |  |
|  |  |  |  |  |  |  |  | |  |
| *Readability (Flesh Kincaid Grade Level)* | .97 |  |  |  |  | 16.03 | 4 | | .003 |
| Easy to read (< or = to 6^th^ grade level) |  | 9 (29) | 9 (29) | 13 (41.9) | 31 (2.8) |  |  | |  |
| Medium to read (between 7^th^ and 9^th^ grade level) |  | 98 (43.9) | 25 (11.2) | 99 (44.8) | 222 (20.4) |  |  | |  |
| Difficult to read (= or > 10^th^ grade level) |  | 407 (48.5) | 74 (8.8) | 359 (42.7) | 840 (76.8) |  |  | |  |
|  |  |  |  |  |  |  |  | |  |
|  |  |  |  |  |  |  |  | |  |
|  |  |  |  |  |  |  |  | |  |
|  |  |  |  |  |  |  |  | |  |
|  |  |  |  |  |  |  |  | |  |
|  |  |  |  |  |  |  |  | |  |
| *b) Vaccination-specific content attributes* | .86 |  |  |  |  |  |  | |  |
|  |  |  |  |  |  |  |  | |  |
| *Type of information* | .85 |  |  |  |  | 6.22 | 4 | | .183 |
| Not able to detect |  | 3 (75) | 1 (25) | - (-) | 4 (0.4) |  |  | |  |
| Detected: single vaccine treated |  | 117 (50.9) | 17 (7.4) | 96 (41.7) | 230 (21) |  |  | |  |
| Detected: more than one vaccine treated |  | 394 (45.8) | 90 (10.5) | 375 (43.7) | 859 (78.6) |  |  | |  |
|  |  |  |  |  |  |  |  | |  |
| *Disease information* | .85 |  |  |  |  | 13.36 | 2 | | .001 |
| Not available |  | 217 (41.8) | 64 (12.3) | 238 (45.9) | 519 (47.4) |  |  | |  |
| Available |  | 297 (51.7) | 44 (7.7) | 233 (40.7) | 574 (52.6) |  |  | |  |
|  |  |  |  |  |  |  |  | |  |
| *Treatment information* | .83 |  |  |  |  | 55.58 | 2 | | < .001 |
| Not available |  | 84 (28.5) | 40 (13.6) | 171 (58) | 295 (27) |  |  | |  |
| Available |  | 430 (53.8) | 68 (8.5) | 300 (37.7) | 798 (73) |  |  | |  |
|  |  |  |  |  |  |  |  | |  |
| *Benefits and risks of vaccination* | .85 |  |  |  |  | 37.92 | 2 | | < .001 |
| Not available |  | 106 (45.7) | 47 (20.3) | 79 (34.1) | 232 (21.2) |  |  | |  |
| Available: |  | 408 (43.7) | 61 (7.1) | 392 (45.6) | 861 (78.8) |  |  | |  |
|  |  |  |  |  |  |  |  | |  |
| Benefits of vaccination | .82 |  |  |  |  | 435.31 | 2 | | < .001 |
| Not available |  | 127 (20.3) | 74 (11.8) | 424 (67.9) | 625 (57.2) |  |  | |  |
| Available |  | 387 (82.7) | 34 (7.3) | 47 (10) | 468 (42.8) |  |  | |  |
|  |  |  |  |  |  |  |  | |  |
| Low risks of vaccination | .84 |  |  |  |  | 9.06 | 2 | | .011 |
| Not available |  | 359 (49) | 80 (10.9) | 294 (40.1) | 733 (67) |  |  | |  |
| Available |  | 155 (42.9) | 28 (7.8) | 177 (49.3) | 360 (33) |  |  | |  |
|  |  |  |  |  |  |  |  | |  |
| Severe risks of vaccination | .93 |  |  |  |  | 331.13 | 2 | | < .001 |
| Not available |  | 411 (72.4) | 53 (9.3) | 104 (18.3) | 568 (51.9) |  |  | |  |
| Available |  | 103 (19.6) | 55 (10.5) | 367 (70) | 525 (48.1) |  |  | |  |
|  |  |  |  |  |  |  |  | |  |
| *Risks of not getting vaccinated ^a^* | .33 |  |  |  |  | 4.17 | 2 | | .124 |
| Not available |  | 297 (44.5) | 68 (10.2) | 302 (45.3) | 667 (61) |  |  | |  |
| Available |  | 217 (50.8) | 40 (9.4) | 169 (39.8) | 426 (39) |  |  | |  |
|  |  |  |  |  |  |  |  | |  |
| *Alternative medicine or treatments* | .81 |  |  |  |  | 52.70 | 2 | | < .001 |
| Not available |  | 478 (51.4) | 91 (9.8) | 360 (38.8) | 929 (85) |  |  | |  |
| Available |  | 36 (22) | 17 (10.4) | 111 (67.7) | 164 (15) |  |  | |  |
|  |  |  |  |  |  |  |  | |  |
| *Definition of terms or (Q&A) format* | .79 |  |  |  |  | 30.56 | 2 | | < .001 |
| Not available |  | 367 (44) | 70 (8.4) | 396 (47.6) | 833 (76.2) |  |  | |  |
| Available |  | 147 (56.5) | 38 (14.6) | 75 (28.8) | 260 (23.8) |  |  | |  |
|  |  |  |  |  |  |  |  | |  |
| *Vaccination Recommendation Schedules (VRS)* | .92 |  |  |  |  | 37.85 | 6 | | < .001 |
| Not available |  | 440 (44.5) | 100 (10.1) | 448 (45.4) | 988 (90.4) |  |  | |  |
| Available: actual (VRS) |  | 73 (73.7) | 6 (6.1) | 20 (20.2) | 99 (9) |  |  | |  |
| Available: past/s (VRS) |  | - (-) | - (-) | 1 (100) | 1 (0.1) |  |  | |  |
| Both actual and past/s (VRS) available |  | 1 (20) | 2 (40) | 2 (40) | 5 (0.5) |  |  | |  |
|  |  |  |  |  |  |  |  | |  |
| *How to get vaccination exemptions legally* | 1.00 |  |  |  |  | 97.85 | 2 | | < .001 |
| Not available |  | 511 (51.3) | 101 (10.1) | 384 (38.6) | 996 (91.1) |  |  | |  |
| Available |  | 3 (3.1) | 7 (7.2) | 87 (89.7) | 97 (8.9) |  |  | |  |
|  |  |  |  |  |  |  |  | |  |
| *Parents (or patients) rights and responsibilities* | .76 |  |  |  |  | 75.89 | 2 | | < .001 |
| Not available |  | 444 (53.6) | 88 (10.6) | 296 (35.8) | 828 (75.8) |  |  | |  |
| Available |  | 70 (26.4) | 20 (7.5) | 175 (66) | 265 (24.2) |  |  | |  |
|  |  |  |  |  |  |  |  | |  |
| *Potential conflict of interests* | .94 |  |  |  |  | 221.22 | 2 | | < .001 |
| Not available |  | 493 (57.7) | 92 (10.8) | 268 (31.5) | 853 (78.1) |  |  | |  |
| Available |  | 21 (8.8) | 16 (6.7) | 203 (84.6) | 240 (21.9) |  |  | |  |
|  |  |  |  |  |  |  |  | |  |
| *Other topics treated* | .73 |  |  |  |  | 13.16 | 2 | | .001 |
| Not available |  | 225 (50.1) | 27 (6) | 197 (43.9) | 449 (41) |  |  | |  |
| Available |  | 289 (44.8) | 81 (12.6) | 274 (42.6) | 644 (59) |  |  | |  |
|  |  |  |  |  |  |  |  | |  |
| *Scope of information* | .91 |  |  |  |  | 260.34 | 6 | | < .001 |
| Not able to detect |  | 1 (5.9) | 3 (17.6) | 13 (76.5) | 17 (1.6) |  |  | |  |
| Detected: general vaccination web site |  | 27 (9.9) | 21 (7.7) | 224 (82.4) | 272 (25) |  |  | |  |
| Detected: specific vaccination web site (e.g., only MMR) |  | 3 (100) | - (-) | - (-) | 3 (0.3) |  |  | |  |
| Detected: general health information web site/ portal |  | 483 (60.3) | 84 (10.5) | 234 (29.2) | 801 (73.2) |  |  | |  |
| *General tone* | .94 |  |  |  |  |  |  | |  |
| Neutral (or undefined) web page |  | - (-) | 108 (100) | - (-) | 108 (9.9) |  |  | |  |
| Pro-vaccination web page |  | 514 (100) | - (-) | - (-) | 514 (47) |  |  | |  |
| Anti-vaccination web page: reformist |  | - (-) | - (-) | 41 (100) | 41 (3.7) |  |  | |  |
| Anti-vaccination web page: radical |  | - (-) | - (-) | 430 (100) | 430 (39.4) |  |  | |  |
|  |  |  |  |  |  |  |  | |  |
| *Links to other vaccination-related web sites* | .92 |  |  |  |  | 6.13 | 2 | | .047 |
| Not available |  | 82 (56.2) | 14 (9.6) | 49 (34.2) | 145 (13.3) |  |  | |  |
| Available |  | 432 (45.6) | 94 (9.9) | 422 (44.5) | 948 (86.7) |  |  | |  |
|  |  |  |  |  |  |  |  | |  |
| *Links to pro-vaccination web sites* | .86 |  |  |  |  | 260.67 | 2 | | < .001 |
| Not available |  | 116 (22.5) | 50 (9.7) | 348 (67.8) | 514 (47.1) |  |  | |  |
| Available |  | 398 (68.7) | 58 (10) | 123 (21.2) | 579 (52.9) |  |  | |  |
|  |  |  |  |  |  |  |  | |  |
| *Links to anti-vaccination web sites* | .91 |  |  |  |  | 633.84 | 2 | | < .001 |
| Not available |  | 493 (77.5) | 64 (10.1) | 78 (12.4) | 635 (58.1) |  |  | |  |
| Available |  | 21 (4.6) | 44 (9.6) | 393 (85.8) | 458 (41.9) |  |  | |  |
|  |  |  |  |  |  |  |  | |  |
| *Links to other online health information resources* | .66 |  |  |  |  | 2.17 | 2 | | .338 |
| Not available |  | 234 (44.7) | 52 (9.9) | 236 (45.3) | 522 (47.8) |  |  | |  |
| Available |  | 280 (49) | 56 (9.8) | 235 (41.2) | 571 (52.2) |  |  | |  |
|  |  |  |  |  |  |  |  | |  |
| ***ONLINE VACCINATION INFORMATION QUALITY (OVIQ)*** | .89 |  |  |  |  |  |  | |  |

NOTE: ^a^ = Discarded variables from the aggregated reliability scores; all the variables are nominal. Sample size: *N* = 1093.
