# Supplementary material for: Comparing the quality of pro- and anti-vaccination online information: a content analysis of vaccination-related webpages
Source: BMC Public Health. 2016 Jan 15;16:38. doi: 10.1186/s12889-016-2722-9 (PMC4714533; doi:10.1186/s12889-016-2722-9)
Supplement: Supplementary file 2 — Comparing the Quality of Pro- and Anti-Vaccination Online Information: A Content Analysis of Vaccination-Related Webpages.pdf. (DOCX 29 kb) [file 12889_2016_2722_MOESM2_ESM.docx]

*Table S3: Webpages’ attributes of pro- and anti-vaccination online information*

|  |  | Pro-vaccination (*N* = 514) | |  | Anti-vaccination (*N* = 471) | |
| --- | --- | --- | --- | --- | --- | --- |
| Variable |  | (*N*) | (%) |  | (*N*) | (%) |
| *Domain name of the website* |  |  |  |  |  |  |
| .com |  | 142 | 27.6 |  | 230 | 48.8 |
| .gov |  | 240 | 46.7 |  | 1 | 0.2 |
| .edu |  | 11 | 2.1 |  | - | - |
| .org |  | 69 | 13.4 |  | 156 | 33.1 |
| .net |  | 2 | 0.4 |  | 23 | 4.9 |
| .info |  | 1 | 0.2 |  | 1 | 0.2 |
| .int |  | 24 | 4.7 |  | 1 | 0.2 |
| .to |  | 2 | 0.4 |  | 58 | 12.3 |
| Other DNS (e.g., .co.uk) |  | 23 | 4.5 |  | 1 | 0.2 |
|  |  |  |  |  |  |  |
| *Functioning of Links* |  |  |  |  |  |  |
| No links available |  | 2 | 0.4 |  | 6 | 1.3 |
| High quality of links |  | 511 | 99.4 |  | 452 | 96 |
| Medium quality of links |  | 1 | 0.2 |  | 6 | 1.3 |
| Poor quality of links |  | - | - |  | 7 | 1.5 |
|  |  |  |  |  |  |  |
| *Provision of more languages than English (yes)* |  | 235 | 45.7 |  | 179 | 38 |
|  |  |  |  |  |  |  |
| *Bar menu (yes)* |  | 444 | 86.4 |  | 359 | 76.2 |
|  |  |  |  |  |  |  |
| *Search toolbar (yes)* |  | 493 | 95.9 |  | 281 | 59.7 |
|  |  |  |  |  |  |  |
| *Interactive tools and services (yes)* |  | 497 | 96.7 |  | 397 | 84.3 |
| E-mail and/or newsletter (yes) |  | 487 | 94.7 |  | 391 | 83 |
| Chat rooms (yes) |  | 3 | 0.6 |  | 1 | 0.2 |
| Forums (yes) |  | 24 | 4.7 |  | 31 | 6.6 |
| Post comments (yes) |  | 59 | 11.5 |  | 150 | 31.8 |
| Other interactive tools or services (e.g., social media buttons) (yes) |  | 339 | 66 |  | 279 | 59.2 |
|  |  |  |  |  |  |  |
| *Privacy policy (yes)* |  | 455 | 88.5 |  | 282 | 59.9 |
|  |  |  |  |  |  |  |
| *Images (yes)* |  | 312 | 60.7 |  | 280 | 59.4 |
| Drugs and medical equipment (yes) |  | 96 | 18.7 |  | 97 | 20.6 |
| Vaccine preventable diseases (yes) |  | 22 | 4.3 |  | 1 | 0.2 |
| People (yes) |  | 216 | 42 |  | 172 | 36.5 |
| Other pictures (yes) |  | 145 | 28.2 |  | 149 | 31.6 |
|  |  |  |  |  |  |  |
| *Graphs/Tables/Diagrams (yes)* |  | 63 | 12.3 |  | 37 | 7.9 |
|  |  |  |  |  |  |  |
| *Videos (yes)* |  | 53 | 10.3 |  | 130 | 27.6 |
| With caregiving profile (yes) |  | 9 | 1.8 |  | 22 | 4.7 |
| With patient (yes) |  | 16 | 3.1 |  | 21 | 4.5 |
| With health professional (yes) |  | 26 | 5.1 |  | 30 | 6.4 |
| Other kind of records (yes) |  | 36 | 7 |  | 77 | 16.3 |
|  |  |  |  |  |  |  |
| Presence of title (*yes)* |  | 511 | 99.4 |  | 464 | 98.5 |
|  |  |  |  |  |  |  |
| *Ownership type (yes)* |  | 487 | 94.7 |  | 369 | 78.3 |
| Government, public or international (yes) |  | 278 | 54.1 |  | 1 | 0.2 |
| Academic (yes) |  | 13 | 2.5 |  | - | - |
| Not-for-profit organization (yes) |  | 69 | 13.4 |  | 265 | 56.3 |
| Commercial (yes) |  | 114 | 22.2 |  | 35 | 7.4 |
| Individual homepage (yes) |  | 13 | 2.5 |  | 68 | 14.4 |
|  |  |  |  |  |  |  |
| *Medical ownership (yes)* |  | 412 | 80.2 |  | 27 | 5.7 |
|  |  |  |  |  |  |  |
| *Purpose of the webpage* |  |  |  |  |  |  |
| Not able to detect |  | - | - |  | 1 | 0.2 |
| Informational or educational (yes) |  | 512 | 99.6 |  | 460 | 97.7 |
| Promotional or commercial (yes) |  | 2 | 0.4 |  | 10 | 2.1 |
|  |  |  |  |  |  |  |
| *Explicit disclosure of target audience (yes)* |  | 129 | 25.1 |  | 43 | 9.1 |
| Patients and/or caregivers (yes) |  | 431 | 83.9 |  | 466 | 98.9 |
| Health professionals (yes) |  | 150 | 29.2 |  | 6 | 1.3 |
|  |  |  |  |  |  |  |
| *Date of creation (yes)* |  | 165 | 32.1 |  | 215 | 45.6 |
|  |  |  |  |  |  |  |
| *Date of last update (yes)* |  | 260 | 50.6 |  | 55 | 11.7 |
|  |  |  |  |  |  |  |
| *References of original contents (yes)* |  | 319 | 62.1 |  | 242 | 51.4 |
|  |  |  |  |  |  |  |
| *Contacts and feedback mechanisms (yes)* |  | 487 | 94.7 |  | 393 | 83.4 |
|  |  |  |  |  |  |  |
| *Accreditations (yes)* |  | 100 | 19.5 |  | 5 | 1.1 |
|  |  |  |  |  |  |  |
|  |  |  |  |  |  |  |
|  |  |  |  |  |  |  |
| *Table 3 (Continued): Webpages’ attributes of pro- and anti-vaccination online information* | | | | | | |
|  |  |  |  |  |  |  |
| *Advertising presence (no)* |  | 364 | 70.8 |  | 229 | 48.6 |
| Clear distinction between contents and Ad. (yes) |  | 146 | 28.4 |  | 135 | 28.7 |
| Unclear distinction between contents and Ad. (yes) |  | 4 | 0.8 |  | 107 | 22.7 |
|  |  |  |  |  |  |  |
| *Complementarity statement (yes)* |  | 421 | 81.9 |  | 265 | 56.3 |
|  |  |  |  |  |  |  |
| *Readability index (Flesh Kincaid Grade Level)* |  |  |  |  |  |  |
| Easy to read |  | 9 | 1.8 |  | 13 | 2.8 |
| Medium to read |  | 98 | 19.1 |  | 99 | 21 |
| Difficult to read |  | 407 | 79.2 |  | 359 | 76.2 |
|  |  |  |  |  |  |  |
| *Type of information* |  |  |  |  |  |  |
| Not able to detect |  | 3 | 0.6 |  | - | - |
| Single vaccine treated |  | 117 | 22.8 |  | 96 | 20.4 |
| More than one vaccine treated |  | 394 | 76.7 |  | 375 | 79.6 |
|  |  |  |  |  |  |  |
| *Disease information (yes)* |  | 297 | 57.8 |  | 233 | 49.5 |
|  |  |  |  |  |  |  |
| *Treatment information (yes)* |  | 430 | 83.7 |  | 300 | 63.7 |
|  |  |  |  |  |  |  |
| *Benefits and risks of vaccines (yes)* |  | 408 | 79.4 |  | 392 | 83.2 |
| Benefits of vaccines (yes) |  | 387 | 75.3 |  | 47 | 10 |
| Low risks of vaccines (yes) |  | 155 | 30.2 |  | 177 | 37.6 |
| Severe risks of vaccines (yes) |  | 103 | 20 |  | 367 | 77.9 |
|  |  |  |  |  |  |  |
| *Alternative medicine or treatments (yes)* |  | 36 | 7 |  | 111 | 23.6 |
|  |  |  |  |  |  |  |
| *Definition of terms or (Q&A) formats (yes)* |  | 147 | 28.6 |  | 75 | 15.9 |
|  |  |  |  |  |  |  |
| *Vaccination Recommendation Schedule (yes)* |  | 74 | 14.4 |  | 23 | 4.9 |
|  |  |  |  |  |  |  |
| *How to get vaccination exemptions legally (yes)* |  | 3 | 0.6 |  | 87 | 18.5 |
|  |  |  |  |  |  |  |
| *Parents (or patients) rights and responsibilities (yes)* |  | 70 | 13.6 |  | 175 | 37.2 |
|  |  |  |  |  |  |  |
| *Potential conflict of interests (yes)* |  | 21 | 4.1 |  | 203 | 43.1 |
|  |  |  |  |  |  |  |
| *Other topics treated (e.g. vaccines for travelers) (yes)* |  | 289 | 56.2 |  | 274 | 58.2 |
|  |  |  |  |  |  |  |
| *Scope of information of the website* |  |  |  |  |  |  |
| Not able to detect |  | 1 | 0.2 |  | 13 | 2.8 |
| General vaccine information website (yes) |  | 27 | 5.3 |  | 224 | 47.6 |
| Specific vaccine information website (yes) |  | 3 | 0.6 |  | - | - |
| General health information web portal (yes) |  | 483 | 94 |  | 234 | 49.7 |
|  |  |  |  |  |  |  |
| *Links to pro-vaccination websites (yes)* |  | 398 | 77.4 |  | 123 | 26.1 |
| *Links to anti-vaccination websites (yes)* |  | 21 | 4.1 |  | 393 | 83.4 |
| *Links to other health information resources (yes)* |  | 280 | 54.5 |  | 235 | 49.9 |

NOTE: all variable are nominal. Sample size: *N* = 985
